# Missing data imputation in hourly CO measurements for air quality monitoring: a case study in the city of Salvador, Brazil

**DOI:** 10.1007/s10661-026-15505-9

**Published:** 2026-05-27

**Authors:** Taiane Campos Braga de Assis, Cristiano Hora Fontes, Édler Lins Albuquerque

**Affiliations:** 1 Programa de Engenharia Industrial (Graduate Program in Industrial Engineering), Escola Politécnica (Polytechnic Institute), Universidade Federal da Bahia (Federal University of Bahia), Salvador, Brazil; 2 Departamento de Processos Industriais E Engenharia Química (Department of Industrial Processes and Chemical Engineering), IFBA—Instituto Federal de Educação, Ciência e Tecnologia da Bahia (Federal Institute of Education, Science and Technology of State of Bahia), Salvador, Brazil

**Keywords:** Time series imputation, Missing data, Machine learning, Air quality monitoring, Urban pollution

## Abstract

Continuous and uninterrupted air quality monitoring is essential for environmental management and public policy formulation, which requires the absence of missing data and good quality measurements. However, due to a variety of factors (local power outages, data transmission, instrument calibration, preventive maintenance, weather conditions, etc.), measurement gaps with different time windows frequently occur in historical air quality data. This work addresses the problem of missing data in air quality monitoring time series, which compromises the quality of information and hinders decision-making related to air pollution. Carbon monoxide (CO) data were imputed in artificially generated gaps (from 24 to 72 h) for a monitoring station located in Salvador, Bahia (Brazil). Three dynamic modeling strategies with different architectures and learning algorithms were applied: XGboost and two recurrent neural networks (LSTM and RNN). The results showed that, although XGboost presented the lowest medians associated with RMSE and MAE distributions (0.1028 and 0.1266 ppm, respectively), the difference compared to the neural networks was not statistically significant. The statistical analysis of the predictions showed that the mean of the residuals does not differ significantly from zero, indicating an absence of systematic bias and suggesting that the imputed values preserve the dominant dynamics and seasonal patterns of the original series. The percentages of gaps consistently described by the models were 82.0% (XGboost) and 91.3% (LSTM and RNN recurrent neural networks). The results demonstrate that the adopted model structures (decision tree and recurrent neural networks), along with a systematic approach involving the analysis and preparation of the training sample (identification of input variables, mapping of existing gaps in the historical data of the measurement station, and generation of artificial gaps, among others), enabled the imputation of dynamic CO data, preserving the dominant behavior of the time series and ensuring the validity of environmental monitoring.

## Introduction

According to the United Nations (UN), cities with more than 500,000 inhabitants should systematically monitor air quality to ensure the control and monitoring of impacts caused by air pollution. Air quality monitoring is necessary for the protection of public health, prevention, and control of pollution, as well as to provide support for the management of environmental policies, the development of early detection systems, forecasting, and decision-making (Li et al., 2023; Pak et al., 2020; Wang et al., 2023; Zhang et al., 2020).

Air quality monitoring data are essentially dynamic (time series) and multivariate in nature. This implies that the frequent occurrence of a lack of continuous measurements over different time intervals (without predictability), involving one or more variables (air pollutants and meteorological variables), represents a loss of information and has a direct effect on environmental monitoring and control. Furthermore, especially when air quality reaches critical levels over short periods, continuous and uninterrupted monitoring is necessary, enabling real-time assessment of potential impacts on public health and compliance with legal requirements. Therefore, the effectiveness of this monitoring depends directly on the completeness (absence of missing data) and quality of the measurements (Zhang & Zhou, 2024). Difficulties include the high cost of implementing and maintaining the stations, the spatial distribution between them, and the need to ensure technical capacity to handle the complexity and heterogeneity of the data resulting from the large volume of information collected (Huang et al., 2021a, b).

According to (Huang et al., 2021a, b), the causes of data loss in air quality monitoring networks are varied. They range from equipment failures and gaps in maintenance and calibration procedures to problems in data transmission, adverse weather conditions, and power outages. These failures compromise the continuous monitoring of pollution rates, hindering enforcement regarding compliance with national air quality standards. The lack of data also compromises retrospective analyses and the application of predictive models, representing a limitation for studies in the field of public health (Kebalepile et al., 2024; Wang et al., 2023).

The distribution of measurement gaps constitutes another limiting factor which directly influences the accuracy of imputation models. The performance of these models can also be affected by other factors, such as the absence mechanism (different gap patterns), the proportion of missing data, and the length of gap windows (Choi et al., 2023). The absence mechanism is related to the observed variables and the probability of measurement failure (Thomas & Rajabi, 2021). The classification of absence mechanisms was proposed by Rubin (1976) and is widely used in several works (Oberman & Vick, 2024; Belachsen & Broday, 2022; Thomas & Rajabi, 2021). The three main mechanisms are as follows: (i) Missing Completely At Random (MCAR), in which the absence is not related to any observed variable nor to the missing value itself; (ii) Missing At Random (MAR), in which the absence depends on observed variables, but not on the missing value itself; and (iii) Missing Not At Random (MNAR), in which the absence depends on the missing value itself (Sun et al., 2023). According to Belachsen and Broday (2022), the mechanism responsible for the absence of data has a great influence on how the gaps are distributed throughout the time series, as well as on the performance of data imputation models. The authors highlight that in cases of air quality monitoring, it is common to assume the Missing At Random (MAR) mechanism.

Regarding the proportion of missing data, Choi et al. (2023) highlight that an increase in the proportion of missing data requires the use of more advanced methods, such as multiple imputation techniques or machine learning (ML)-based approaches. Furthermore, the larger the time interval of the gap, the lower the ability of imputation algorithms to identify long-term patterns in time series. When analyzing the imputation of water level telemetry data in reservoirs, Khampuengson et al. (2023) found that the performance of different dynamic modeling techniques is directly associated with the size of the gap.

In the event of operational failures, such as sensor malfunctions, power outages, or extreme weather conditions, validated dynamic models can be activated to maintain the continuity of time series. Predictive empirical models are applied in different sectors and types of problems (e.g., urban traffic, identification of critical pollution episodes, and support in maintenance strategies and station calibration) (Zhang et al., 2020; Belachsen et al., 2022; Narasimhan & Vanitha, 2023; Wang et al., 2023; Li et al., 2021; Cabaneros et al., 2019). In the specific case of data imputation in historical series, predictive models are important to ensure the reliability of real databases and the efficient management of monitoring networks.

In the field of air quality in particular, several authors have investigated imputation methods focusing on restoring data completeness and preserving temporal patterns. The study conducted by Betancourt et al. (2023) focused on the imputation of tropospheric ozone data using a machine learning algorithm. The authors used hourly observations from 278 stations throughout 2011. The ozone data were combined with geospatial metadata, meteorological data, and reanalysis data. The proposed methodology comprised a combined strategy involving linear interpolation for short gaps (up to 5 h) and a Random Forest model for gaps with longer periods. Belachsen and Broday (2022) presented an approach based on a multivariate KNN model, which also considered past and lagged observations. Data from 59 air quality monitoring stations were collected between 2012 and 2019. The proposed model showed a significant improvement in prediction for very short gaps $\left(\le 3h\right)$ and similar performance to the reference method for larger ones. Yu et al. (2023) applied the hierarchical retrieval method using an LSTM network for the retrieval of missing air pollutant data in Hong Kong. Kebalepile et al. (2024) evaluated the effectiveness of the multiple chain equation imputation (MICE) algorithm in the retrieval of missing data with random mechanism (MAR). The work involved the use of data from several monitoring stations, including daily averages of NO_2_, SO_2_, O_3_, and PM_10_, collected between 2010 and 2017. Zhang and Zhou (2024) developed a study aimed at filling continuous long-term gaps in time series of air pollution. The research was conducted in a city in Shaanxi province, China, using data from 225 stations, with hourly records of PM_2.5_, PM_10_, SO_2_, NO_2_, CO, and O_3_, collected between 2018 and 2020. The proposed model, TMLSTM-AE (Transferred Spatio-temporal Deep Model based on Multi-LSTM Auto-Encoder) combined autoencoder transfer learning and LSTM network.

This paper presents a systematic approach for imputing missing data from air pollution monitoring stations. The proposed approach involved comparing results obtained from three classical prediction models (a decision tree-based model, conventional short-term recurrent neural network and recurrent neural network with gate-based neuron model) and the results were validated through a real-world case study (imputation of carbon monoxide concentration data from an air quality monitoring station in the city of Salvador, Brazil). The following contributions are associated with this work:

- In addition to developing the forecasting models themselves, this work involves essential steps in preliminary problem analysis, including identifying predictor variables; recognizing seasonal patterns and similarities between variables; and characterizing the frequency, duration, and temporal distribution of gaps (absence of measurements) in the available database.
- An integrated methodology is therefore proposed for data imputation in air quality monitoring stations, capable of identifying the intrinsic aspects of the problem (input variables, non-contemporaneous correlation between inputs and outputs, and patterns of features among the gaps), improving the performance of the models used to predict missing data.

## Methodology

### Database and study region

The database used in this study was provided by CETREL (Environmental Protection Company Inc., Camaçari Industrial Complex, Bahia, Brazil), which managed the air quality monitoring network for the city of Salvador between 2011 and 2016. The monitoring network consisted of 8 fixed stations, distributed in different neighborhoods of the city, providing data on hourly average concentrations of air pollutants (SO_2_, PM, CO, NO, NO_2_, and NO_*x*_) and meteorological variables (temperature, relative humidity, wind speed, and wind direction). In this work, the variable selected as the target for data imputation was CO. The choice of CO as the target variable is justified by the fact that it is a primary pollutant, mainly emitted by mobile sources resulting from the incomplete combustion of fossil fuels in urban atmospheres. This feature is consistent with the proximity of the Salvador monitoring network stations to traffic routes, which measures the significant effect of motor vehicles as sources of emission of this pollutant in the monitored locations. Despite the importance of CO monitoring, a high frequency of gaps was observed in the measurements of this pollutant at all stations, making the development of an imputation model a challenge for the continuous monitoring of air quality.

**Fig. 1:**
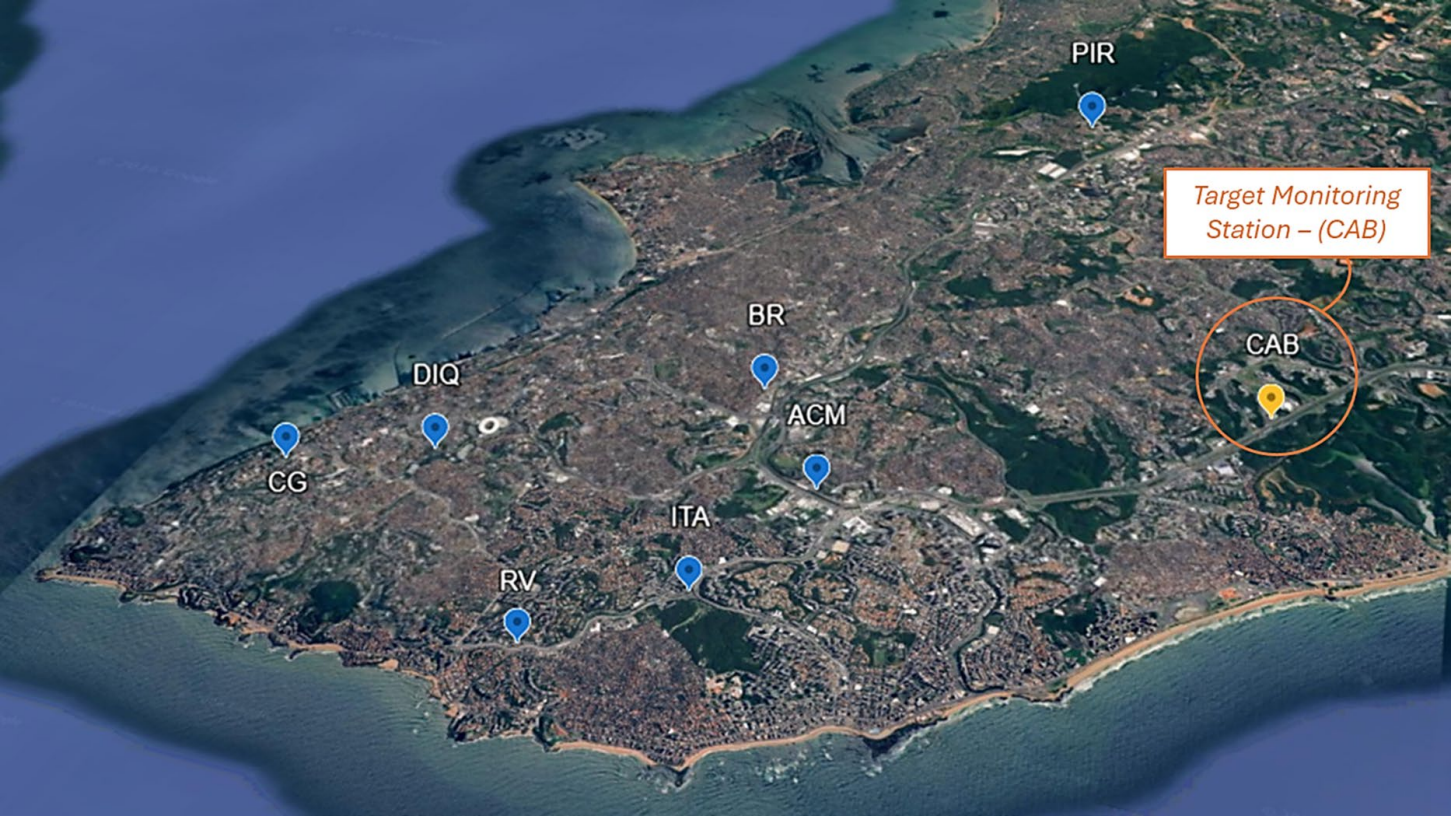
Location of air quality monitoring network stations in the city of Salvador and identification of the target station (CAB)

The data used in this work refer to one of the 8 existing stations, with local characteristics that directly influence the dynamics of air pollution, such as topography and emission sources. The chosen station was located on the side of a high-traffic road. Figure 1 shows the location of the monitoring network stations in the city of Salvador and the location of the target station chosen for this study (the State of Bahia Administrative Center—CAB—station). The CAB station was located near an avenue with heavy vehicle traffic and was the first monitoring unit installed in the city of Salvador (December 2010). This station was selected due to the greater availability of measurements for all variables investigated, thus allowing for a more comprehensive assessment of the monitoring history. The CAB station was positioned at UTM coordinates $E=562000 m$ and $N=8567903 m$, in the SAD69 reference system, zone 24S.

### Definition of the input (predictor) variables

Initial exploratory analyses were carried out to define input (exogenous) variables. Given the essentially multivariate and dynamic nature of the problem, the main objective of this analysis was to support the selection of predictor variables. A cross-correlation analysis was performed considering positive lags $\left(\tau =0, 1, 2, \dots , 25\right)$ (each lag is equivalent to 1 h). The use of only positive lags is aimed at analyzing the intensity of the causal relationship of each of the other observed variables (inputs) (SO_2_, PM, CO, NO, NO_2_, NO_*x*_, temperature, relative humidity, and wind speed) on the CO concentration (output variable) at different time delays.

**Fig. 2:**
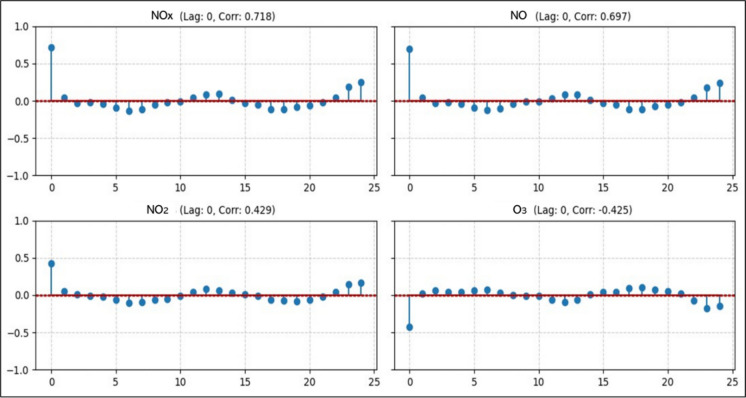
Cross-correlation between CO and Predictor Variables after Differentiation

Due to the non-stationary nature of time series, the first-order differentiation technique was adopted. The original series was transformed into a new series with variations in the original measurements at consecutive instants, allowing for the isolation of short-term fluctuations and avoiding spurious correlations. The cross-correlation function (positive lags) was applied to the stationary series, generating correlograms (Fig. 2) for each of the possible inputs (exogenous variables) with CO. As shown in Fig. 2 (e.g., NO_*x*_, NO, NO_2_, and O_3_), the strong correlation observed at lag zero indicates that the relationship between the predictor variables and the CO concentration is predominantly simultaneous, justifying the use of the data measured at the same time instant as the main predictor in the models. It is important to emphasize that this feature is directly related to the sampling period adopted (1 h).

### Simulation scenarios

Validating a prediction model using existing gaps in the database is unfeasible due to the absence of measured data. Therefore, it was necessary to create a test scenario that involved generating synthetic gaps by excluding measurements from the original database. In turn, these same synthetic gaps were not part of the model's training sample and were used as a test and validation sample for the imputed series, which enabled the calculation of performance metrics (Mean Absolute Error—MAE; Root Mean Square Error—RMSE).

On the other hand, the generation of synthetic gaps for testing/validating the model must be carried out in accordance with the pattern of the real gaps existing in the database, which implies identifying the periods (time window lengths) and their respective frequency distribution. After capturing all the duration periods of the gaps existing in the database, a frequency distribution was generated assuming four duration ranges: (i) short gap (1 to 24 h), (ii) medium gap (25 to 72 h), (iii) long gap (73 to 120 h), and (iv) very long gap (121 to 168 h). Table  1 95% confidence intervalsModel95% CI—MAE (ppm)Margin of error—MAE (ppm)95% CI—RMSE (ppm)Margin of error—RMSE (ppm)XGboost(0.1037–0.1269) ± 0.0116(0.13071–0.1599) ± 0.0146LSTM(0.1122–0.1663) ± 0.0271(0.1352–0.1953) ± 0.0301RNN(0.1134–0.1487) ± 0.0176(0.1394–0.1818) ± 0.02121 presents a summary of the distribution of the number of gaps by duration interval.

**Table 1:** Distribution of gaps by duration range

Duration range	Number of gaps	Average duration (h)	Maximum duration (h)
Short gap (1–24 h)	72	2.6	22.0
Medium gap (25–72 h)	3	37.0	47.0
Long gap (73–120 h)	1	82.0	82.0
Very long gap (121–168 h)	0	0.0	0.0

As shown in Table 1, the duration of the actual gaps is mostly concentrated in the range of 1 to 24 h. Considering that predicting shorter gaps in CO concentrations involves a less complex imputation problem, which can be solved with simpler methods, and that the occurrence of long-duration gaps is not verified, the range of 25 to 72 h was adopted as the target range for validating the prediction models. Although less frequent than the short duration, the range of 25 to 72 h is of operational importance as it corresponds to the average period practiced for maintenance and calibration operations of the monitoring stations (about 1 to 3 days). Thus, the test scenario, composed of synthetic gaps, was configured to evaluate the performance of the models for imputing CO concentrations considering prediction periods of 25 to 72 h, with predictions at each hourly interval.

The training sample was generated using a sliding window approach, in which, for each instant, a 48-h period prior to the target variable (CO concentration) was considered. Only time points with complete data were used, excluding those belonging to artificial gaps and those without measurements of CO concentration or exogenous variables. This procedure resulted in the generation of 33,830 multivariate time series (exogenous variables and CO concentrations) which constituted the training sample. The test sample was used to validate the prediction models, which in turn were identified from the training sample.

A test dataset was generated (a copy of the original dataset) with artificial gaps of random sizes within the 25- to 72-h interval. The artificial gaps were generated in the dataset during periods when the original time series was fully complete, thus avoiding overlap between artificial and real gaps. This procedure ensures the existence of CO concentration measurements, enabling the validation of the trained models’ performance using consolidated metrics for regression problems. Therefore, there was no overlap between the artificial and real gaps, and the artificial gaps were generated in such a way that they did not overlap each other, which is necessary to avoid redundancy in the results and ensure that the models were tested at different periods throughout the entire time series. All artificial gaps were inserted into the test sample, ensuring that the prediction models obtained using different methods were compared based on the same prediction horizons. A total of 46 test gaps were generated. The artificial gaps were used exclusively to evaluate the model’s performance.

### Imputation models and validation

**Fig. 3:**
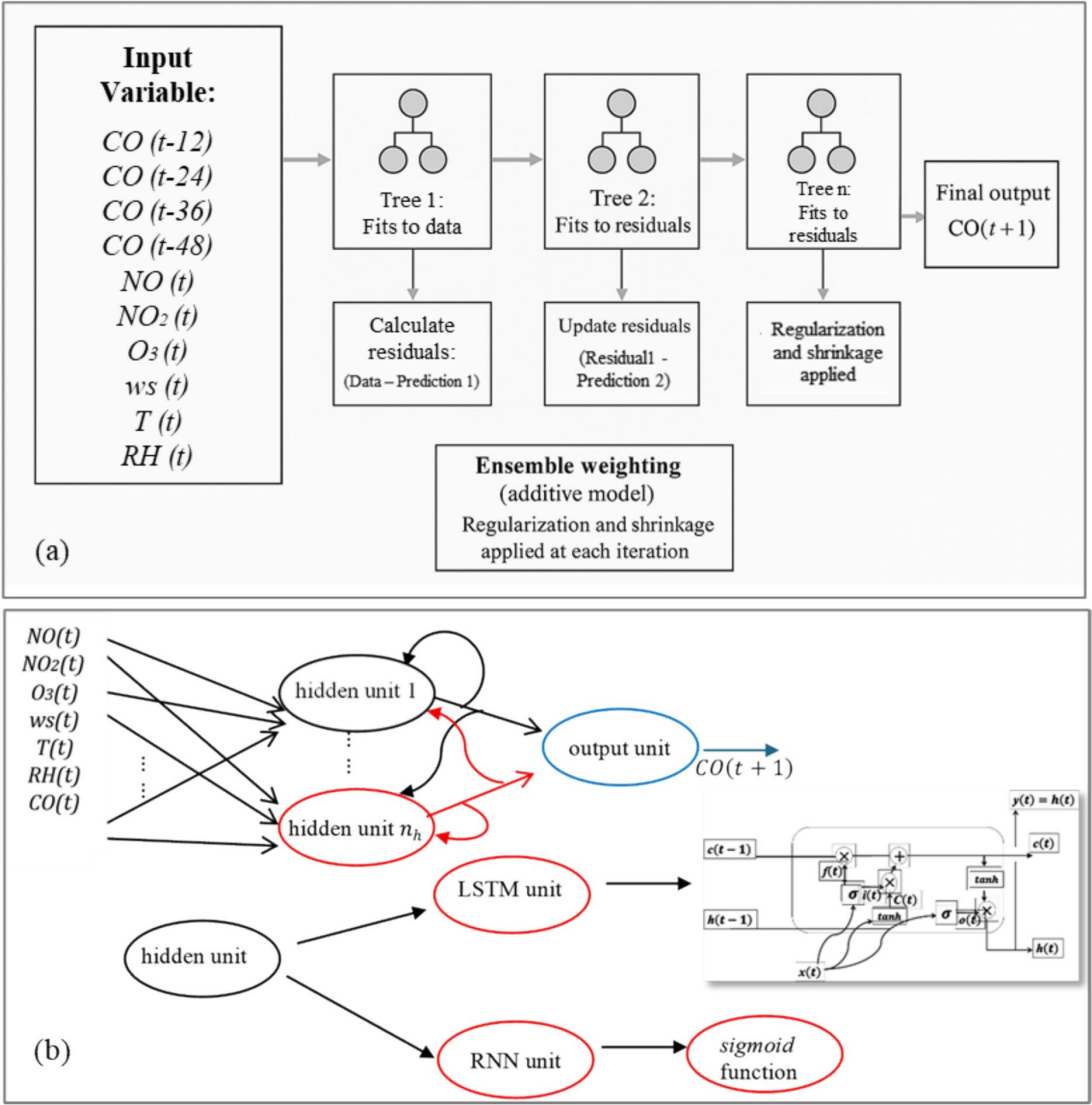
Architecture of the models used in CO data imputation—XGBoost (**a**) and neural network models (RNN and LSTM) (**b**)

Three model alternatives with different architectures and learning (training) strategies were considered, all capable of performing long-range prediction (multiple steps ahead from the initial instant) of time series involving exogenous variables (inputs) with an effect on the output (CO concentration). The approaches considered were: XGBoost (Extreme Gradient Boosting)—a decision tree-based model capable of capturing complex non-linear patterns, and which represents a benchmark technique among classical machine learning-based regression approaches (Fig. 3a); Simple RNN (Recurrent Neural Network)—recurrent neural networks with a conventional neuron model (non-linear activation function), complete feedback of hidden states, and short-term memory capability; and LSTM (Long Short-Term Memory)—recurrent networks that use hidden units (neurons) with a gate-based structure (input, forget and output gates) whose main feature is the ability to recognize long-term dependencies and patterns (Fig. 3b). The use of LSTM networks in this work also enabled an assessment of the existence of “long memory” in cause-and-effect relationships and its impact on the performance of the predictive model considering prediction horizons between 24 and 72 h. The three models comprise a diverse set of options, considering that one of them (XGBoost) consists of a static structure and is based on a decision tree, while the others comprise an essentially dynamic structure (recurrent networks) with differences in the ability to store long-term memory.

The configurations (number of hidden layer, number of units, and activation functions) adopted in both neural networks (RNN and LSTM) are similar (Table 2), although the LSTM network required a smaller number of units. In both models, the Mean Square Error (MSE) was adopted as the loss function along with Adam’s optimization method, widely employed in other works involving dynamic networks (Makinde, 2024; Solgi et al., 2021). In the XGBoost model, the input vector comprised the output variable $\left(CO\right)$ at 4 past instants $\left(CO\left(t-12\right), CO\left(t-24\right), CO\left(t-36\right), CO\left(t-48\right)\right)$ and four exogenous variables measured at the present instant (NO_2_, NO, temperature, and relative humidity). Dynamic information is learned exclusively by the CO lags, since XGBoost does not have internal memory mechanisms. The adopted configuration included 600 trees, a maximum depth of 6, a learning rate of 0.05 and the MSE loss function. In the data imputation stage, prediction is performed only when all necessary lags are available, which limits performance during prolonged periods of data absence, especially in the absence of multiple consecutive time periods.

**Table 2:** LSTM and RNN neural networks

	LSTM	RNN
First hidden layer	64 LSTM units	128 RNN units (sigmoid function)
Second hidden layer	32 LSTM units	64 RNN units (sigmoid function)
Third hidden layer	64 units (ReLU function)	128 units (ReLU function)

The following exogenous variables (inputs or predictors) were considered based on the results obtained through cross-correlation analysis (Section "Definition of the input (predictor) variables"): nitrogen dioxide (NO_2_), nitric oxide (NO), ozone (O_3_), temperature, relative air humidity, and wind speed—all measured at the target monitoring station in this work and at the same time as the carbon monoxide (CO) measurement.

Considering that the exogenous variables (and the CO concentration itself) do not have well-defined upper and lower limits, all data were pre-processed using *z*-score normalization. The transformation of the predictions (CO concentrations) to the original unit of measurement (ppm) was performed based on the mean and standard deviation obtained for the training sample (2011 to 2016).

Unlike RNN and LSTM networks, the XGBoost model required the inclusion of lagged variables (delays of 12, 24, 36, and 48 h) in the data preparation stage to explicitly represent the temporal dependence of the CO series, since its architecture does not incorporate hidden state dynamics. In the case of neural networks, training was performed considering sliding windows of 48 h, and the network was trained to predict the CO concentration at the next instant. Although the models were trained for one-step-ahead prediction, they were validated with the test sample (artificial gaps) considering long-range prediction (multiple steps ahead), which is consistent with the features of the problem.

The model validation analysis comprised the application of metrics that penalize higher prediction errors (Mean Absolute Error—MAE; Root Mean Square Error—RMSE), the Coefficient of Determination (*R*^2^), which assesses the proportion of variance explained by the models, and a set of hypothesis tests (parametric and non-parametric). Initially, the assumption of normality of the error distributions (MAE and RMSE) and the coefficient of determination (*R*^2^) for each model was evaluated using the Shapiro–Wilk test (Costa et al., 2021; Fontes & Embiruçu, 2021). Non-parametric tests were performed when the normality assumption was not met for all distributions $\left(p<0.05\right)$. The comparison between the three models was performed based on the Kruskal–Wallis test (Costa et al., 2021; Fontes & Embiruçu, 2021), also considering 95% confidence intervals (95% CI) for the mean of each performance metric. The interpretation of these tests in the results section follows the standard convention in which a *p-value* less than 0.05 indicates a statistically significant difference and small confidence intervals $\left(\pm 5-15\%\right)$ suggest greater precision and reliability of the estimates (Costa et al., 2021; Fontes & Embiruçu, 2021; Huang et al., 2021a, b).

Additionally, the statistical consistency of the imputations was evaluated by analyzing the residuals (the difference between observed and imputed values), adopting a sequence of tests that verify both temporal independence and the presence of systematic bias. Initially, the Ljung-Box test was applied to each gap so as to identify the presence of serial autocorrelation in the residuals (Yenkikar et al., 2025; Zolghadr-Asli et al., 2021). This test starts from the null hypothesis that the residuals are independent $\left(p\ge 0.05\right)$, which would indicate the absence of autocorrelation and, therefore, that the model adequately captured the temporal structure of the data. For the gaps whose residuals did not show significant autocorrelation, the paired t-test was performed, which compares the means of the original and imputed series. In this case, the null hypothesis establishes that the mean of the residuals is equal to zero. Thus, a $p$-value≥0.05 indicates an absence of mean bias, meaning that the model does not tend to overestimate or underestimate CO concentrations.

If the Ljung-Box test indicates temporal dependence ($p$-value<0.05), autocorrelation tests on the residuals should be applied to ensure the validity of the inference. In this case, the Newey–West estimator (HAC) was adopted, which adjusts the standard errors to correct for autocorrelation and heteroscedasticity (Datta & Du, 2012) and the Block Bootstrap method (Önöz & Bayazit, 2012), based on resampling by moving blocks, which preserves the temporal structure of the residuals by empirically estimating the distribution of the mean. For each gap, the final *p*-value was the larger of the two tests (conservative approach) in order to minimize the risk of spurious detection of bias. This combined strategy ensures that the evaluation of imputation models is statistically consistent for both series with independent residuals and those with residual temporal dependence.

**Fig. 4:**
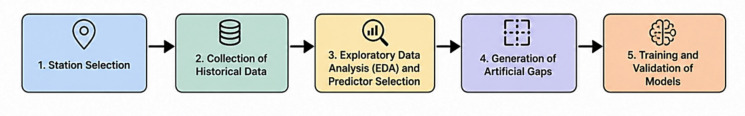
Simplified flowchart of the methodological workflow

The methodological steps adopted in this study are summarized in Fig. 4. The workflow comprises five main stages: (1) selection of the monitoring station, (2) collection of historical data, (3) exploratory data analysis and predictor selection, (4) generation of artificial gaps, and (5) training and validation of imputation models. This sequence was designed to ensure that models are trained and evaluated under conditions that reflect real-world scenarios while preserving the integrity of the original data.

## Results and discussion

The analysis of the results and evaluation of the models (XGBoost, RNN, and LSTM) were based on performance metrics (MAE, RMSE, and *R*^2^) and on a comparative visual analysis between the approaches.

**Fig. 5:**
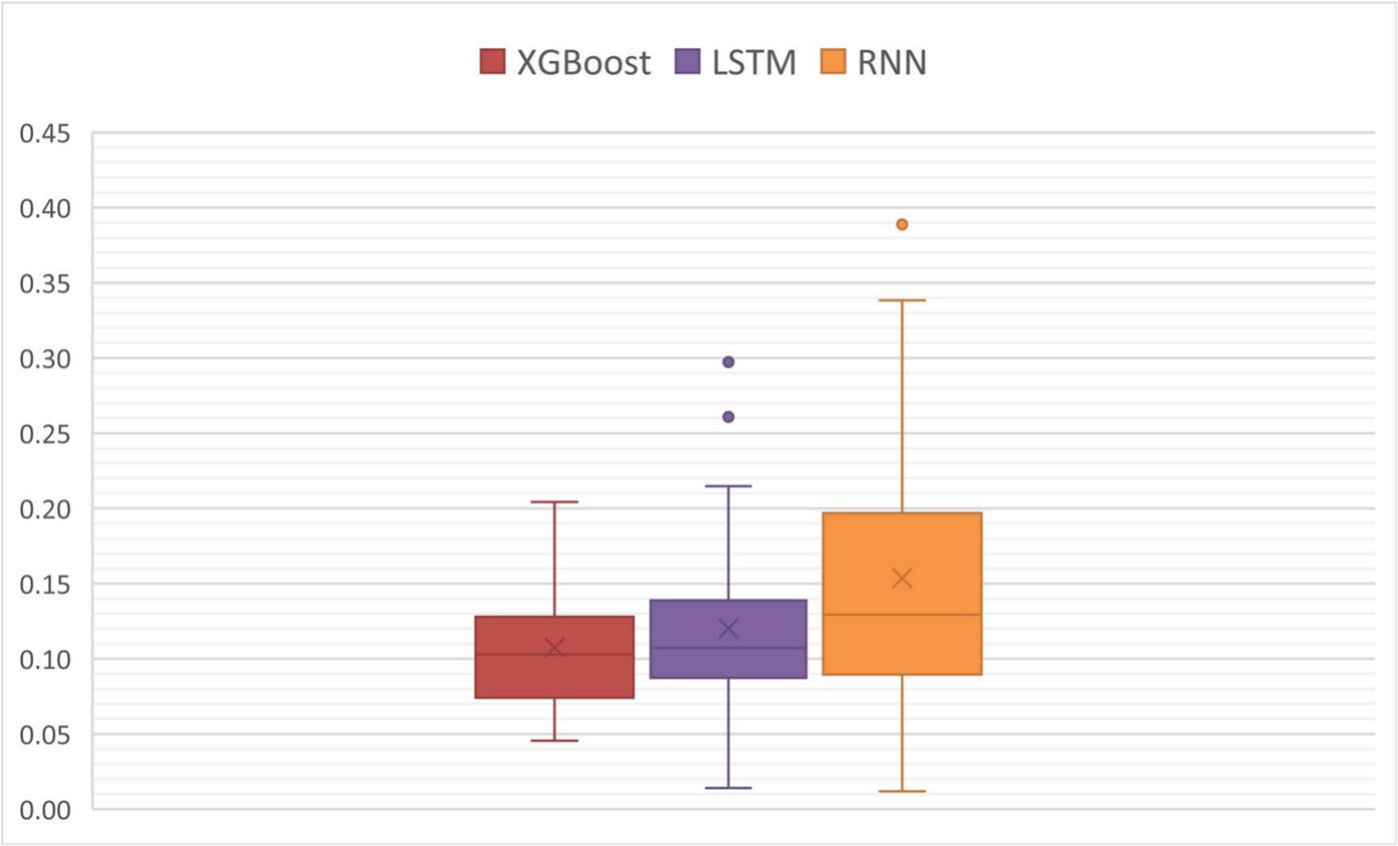
Distribution of mean absolute error (MAE) (ppm) in artificial gaps

**Fig. 6:**
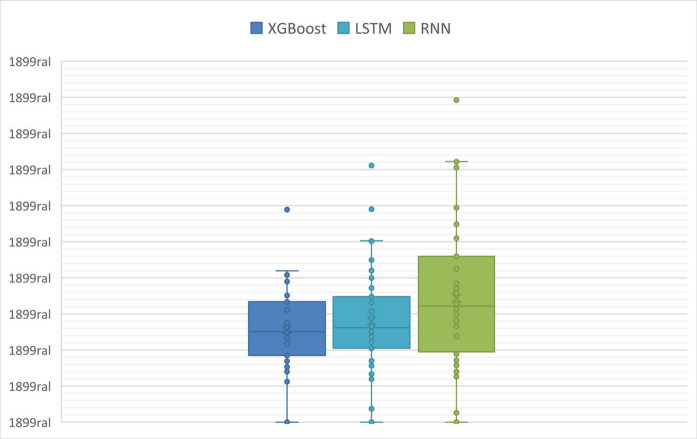
Distribution of the root mean square error (RMSE) (ppm) in artificial gaps

A first analysis comprised verifying the distribution of MAE and RMSE metrics across the entire test sample (all artificial gaps) in each model (XGboost, RNN, and LSTM) (Figs. 5 and 6). The box-plot analysis shows that the XGboost and LSTM models perform quite similarly, not only in terms of medians (MAE distributions equal to 0.1028 (XGboost) and 0.1072 (LSTM), and RMSE distributions equal to 0.1266 (XGboost) and 0.1310 (LSTM)), but also in terms of the dispersion of the distributions (height of the boxes), which demonstrates less variability in their performance when applied to different gap sizes (different prediction horizons). This suggests greater robustness of these models and the ability to impute data in different scenarios while maintaining consistency in prediction errors.

On the other hand, the RNN model (median of the MAE and RMSE distributions equal to 0.1294 and 0.1637, respectively) showed greater sensitivity regarding its application in different scenarios (different prediction horizons) and is therefore more dependent on specific features of each measurement gap.

**Fig. 7:**
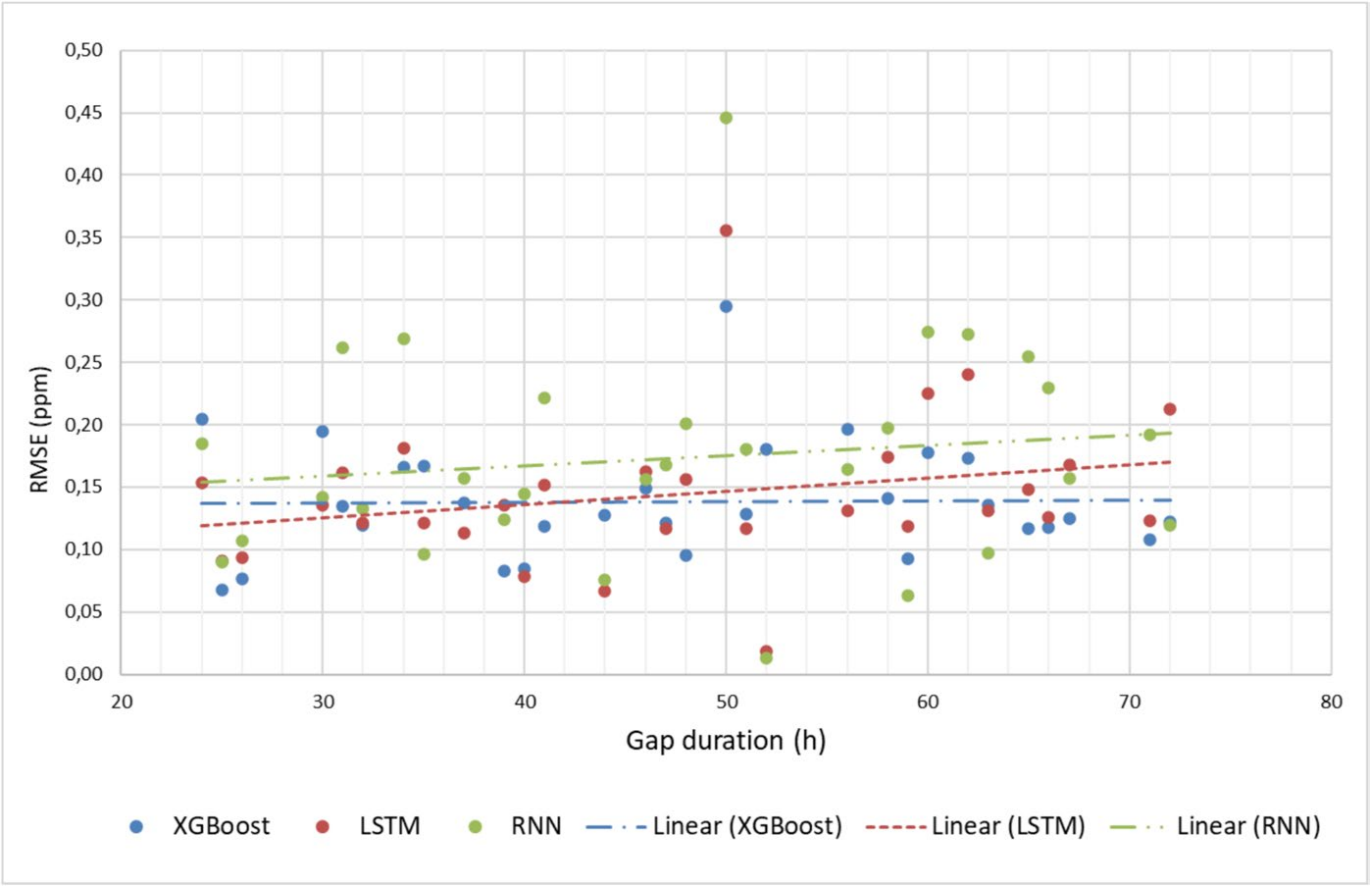
RMSE (ppm) of the models for different prediction horizon sizes

Figure 7 presents RMSE values obtained by the 3 models for different measurement gap lengths (prediction horizons) and their respective linear fit. In general, the XGboost and LSTM models continue to show better performance compared to the RNN, although the XGboost model showed a more stable RMSE behavior in relation to the increase in gap size.

**Fig. 8:**
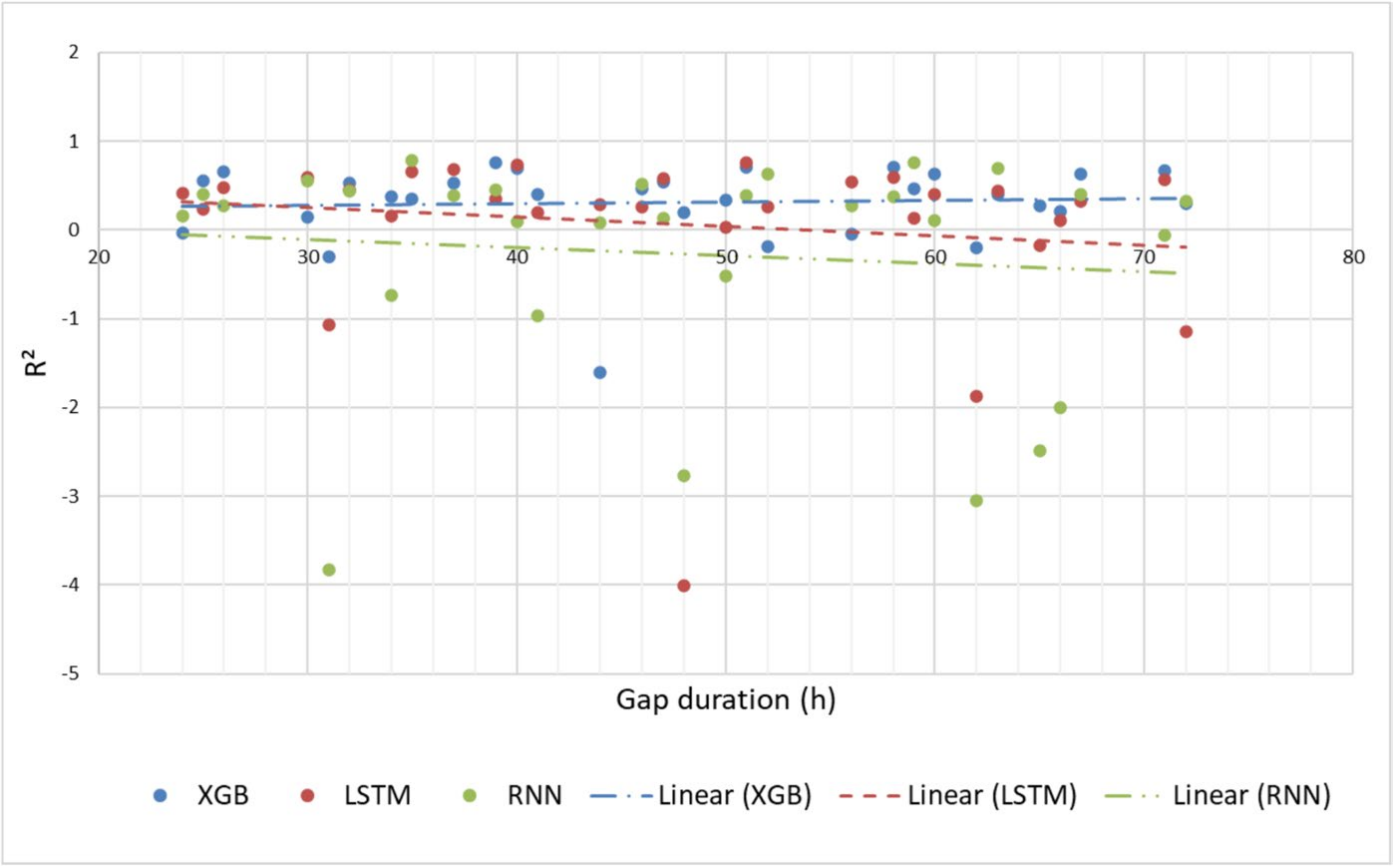
*R*^2^ based on the duration of the gap

Regarding the coefficient of determination (*R*^2^), it is also observed that the XGboost model presents a more stable performance as a function of the increase in the size of the prediction horizon, suggesting in general that this model is able to explain a greater proportion of the variance in the data, even with the increase in the size of the measurement gaps. In some scenarios, negative R^2^ values were obtained by all models, which suggests, in these cases, the existence of other exogenous effects on the CO concentration not considered in the modeling. The LSTM model presented a lower frequency of negative *R*^2^ values (Fig. 8).

The results obtained using the Kruskal–Wallis (Lee et al., 2024, Terry & Shakyra, 2024) test showed that for all the metrics evaluated (MAE, RMSE, and *R*^2^), the differences between the distributions were not statistically significant ($p>0.05$), which implies that none of the models consistently outperformed the others from a statistical point of view. In practical terms, this means that although the average errors suggest variations between methods, these differences cannot be generalized to the population of possible cases.

**Fig. 9:**
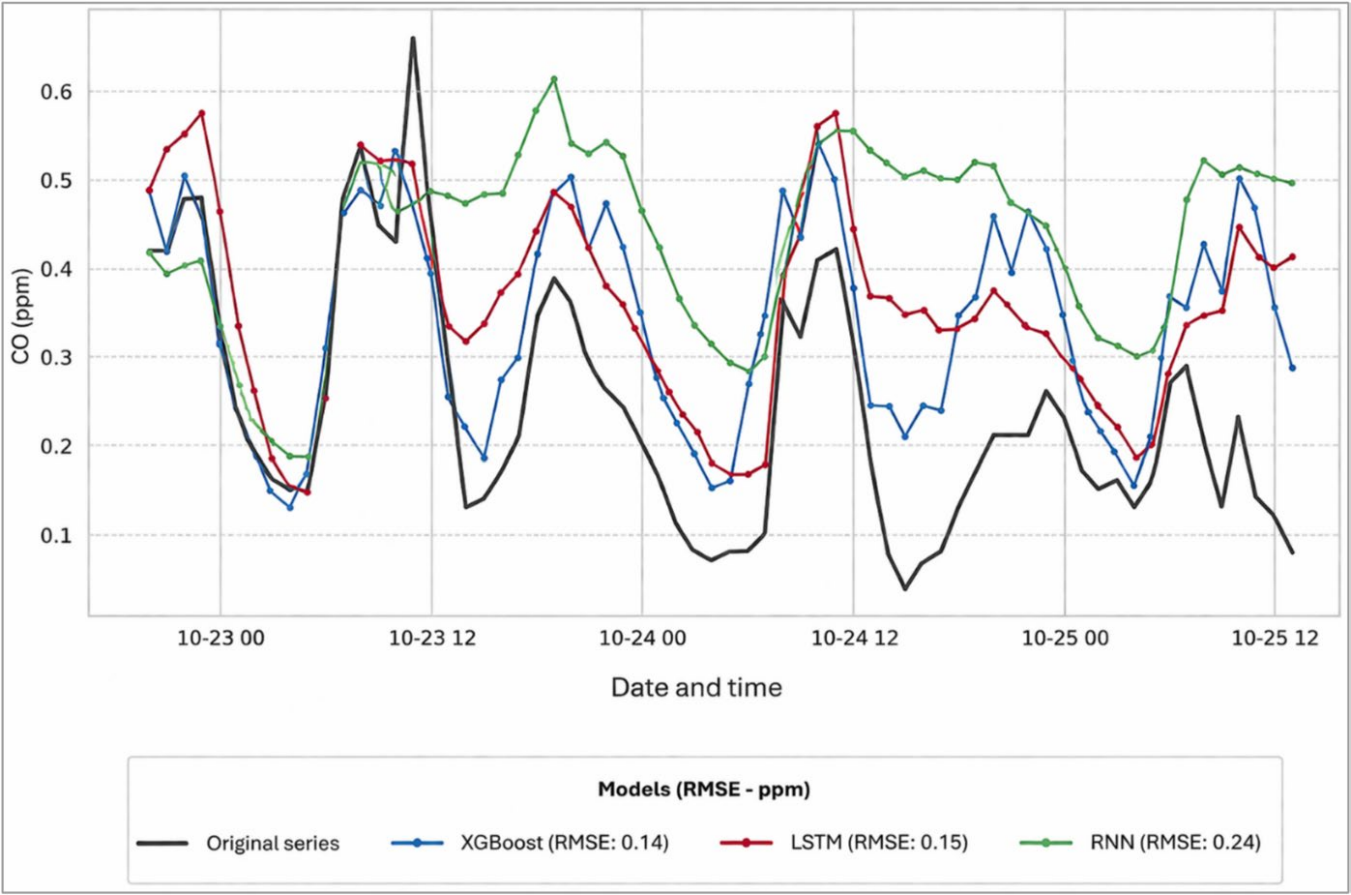
Imputation in gap no. 25 – period 2015-10−22 20 h to 2015-10−25 13 h (65 h)

Figure 9 presents the predictions obtained by the 3 models for a specific case (gap number 25, total duration of 65 h). The results show that the XGboost and LSTM models provide a better description of the dominant dynamics of CO concentration behavior, reproducing the sequence of peaks and valleys of the original series. In contrast, imputation using the RNN approach, while following the general trend, tends to attenuate the magnitude of fluctuations that occur in short periods, resulting in a smoother series, which is consistent with its higher RMSE. Overall, this behavior was repeated for the other gaps.

Table 3 presents the confidence intervals – CI 95% confidence intervals (CI) for the MAE and RMSE metrics in all three identified models and their respective deviations from the estimated mean values. In both cases (MAE and RMSE), the XGboost approach presented a more compact confidence interval compared to the models based on recurrent neural networks (LSTM and RNN). Overall, it is reasonable to state that the sample size (46) is sufficient to generate reliable and stable estimates of the model parameters, within an acceptable margin of error.

**Table 3:** Confidence intervals (95%)

Model	CI (95%) – MAE(ppm)	Margin of error—MAE (ppm)	CI (95%) – MAE(ppm)	Margin of error—RMSE (ppm)
XGboost	(0.1037–0.1269)	± 0.0116	(0.13071–0.1599)	± 0.0146
LSTM	(0.1122–0.1663)	± 0.0271	(0.1352–0.1953)	± 0.0301
RNN	(0.1134–0.1487)	± 0.0176	(0.1394–0.1818)	± 0.0212

In addition to performance metrics, the statistical consistency of the predictions (imputations) obtained by the models in all analyzed gaps (test sample) was evaluated through statistical tests based on the mean of the residuals $\left(\mathrm{m}\mathrm{e}\mathrm{a}\mathrm{s}\mathrm{u}\mathrm{r}\mathrm{e}\mathrm{d}-\mathrm{e}\mathrm{s}\mathrm{t}\mathrm{i}\mathrm{m}\mathrm{a}\mathrm{t}\mathrm{e}\mathrm{d}\right)$ not differing significantly from zero. In gaps without autocorrelation of the residuals, the paired t-test was applied and in gaps with autocorrelation, the Newey-West and Block Bootstrap tests (Yang et al., 2024, Wesseling et al., 2024) were applied. In general, these tests verify whether the models do not present systematic mean bias in the imputation of CO data. The joint application of the Newey-West and Bootstrap tests revealed that, even in situations where there was autocorrelation in the residuals (i.e., a significant effect of past errors on the current error), the mean of the residuals remained statistically indistinguishable from zero. This evidence is particularly important because it demonstrates that the observed temporal dependence does not translate into directional bias, ensuring that the imputed values preserve the average behavior of the original series. The percentages of unbiased gaps (82.0% for XGboost and 91.3% for both neural network-based models, LSTM, and RNN) reinforce the robustness of the identified models.

In recurrent neural networks (LSTM and RNN), the presence of autocorrelation in the residuals is not a limitation of the model, but reflects the sequential nature of these architectures, which learn and explicitly reproduce the temporal dependencies of the series. Therefore, the correlation between adjacent errors is expected and consistent with the internal memory mechanism of these model structures, unless this implies significant average bias. The absence of average bias, even under autocorrelation of the residuals, confirms that the models adequately captured the seasonal dynamics and CO variation patterns, without introducing distortions into the dominant trend of the time series.

The bias analysis confirms the statistical consistency and reliability of the imputations performed. This finding is crucial because it ensures that the remaining error in the models does not compromise the integrity of the CO concentration estimates. The use of the models as support tools in measurement stations for predicting missing data is therefore justified.

Regarding the problem of data imputation in a real monitoring station, obtaining negative *R*^2^ values (Fig. 8) (in all prediction models) reveals that, even applying an initial exploratory data approach, the presence of exogenous effects (unmeasured or even unknown) must be considered in data imputation, leading to a loss of physical consistency in the predictions. Specifically for the monitoring station analyzed, the absence of data related to vehicle flow on the avenue near the station and solar radiation may contribute to the drop in performance of the prediction models.

Another aspect that should also be considered is the existence of slow dynamics (high time constant and dead time) in the behavior of CO concentration. This intrinsic feature of the real system justifies the use of LSTM neural networks since, as discussed in section "Imputation models and validation", the processing units (neurons) in this structure are based on different gate models (forgetting, input and output) which allow the capture and maintenance of past information (long memory).

## Conclusions

Based on the results, it can be concluded that there were no statistically significant differences between the three models, although the XGboost model demonstrated better overall performance. The LSTM neural network proved to be a model as robust and consistent as XGboost. While no statistically significant differences were found between the models, the conventional recurrent network (RNN, without the use of hidden units) showed inferior performance in reproducing the sequence of peaks and valleys of the original series, attenuating the magnitude of fluctuations that occur in short periods.

The very similar performance between the XGboost and LSTM models is related to their structural features and the ability to learn long-term memory within cyclical patterns. The initial exploratory analysis suggested the inclusion of inputs in the XGboost model with specific autoregressive lags (12 h, 24 h, 36 h, and 48 h), capable of capturing the daily seasonality of the pollutant. In the case of the LSTM approach, the importance of these lags was recognized from the feeding of a sliding sequence (time window) of 48 h and the intrinsic capacity for long-term memory learning enabled through the gate-neuron model.

Hypothesis tests confirmed that the imputation models preserved the mean of the original CO series, without a systematic tendency to overestimate or underestimate. Even in gaps with autocorrelated residuals, robust Newey-West and Bootstrap tests indicated that the mean of the errors was statistically zero, demonstrating neutrality in the imputations. This finding demonstrates that the models, especially the LSTM and RNN neural networks, captured the temporal dynamics without introducing distortions in the mean value of the concentrations. Thus, the imputations were considered statistically consistent and reliable, ensuring that the reconstructed data maintained the integrity necessary for predictive analyses and air quality monitoring.

The quality of the results obtained confirms the predictive capacity of the models and their statistical consistency as a tool for imputing sound data in a real air quality monitoring station. Additionally, regardless of the approach used (XGboost, LSTM, and RNN), the performance of the identified models is not solely a consequence of the training phase and the definition of appropriate hyperparameters. This work also proposes a systematic approach for exploratory data analysis, analysis of existing gaps, preparation of training and test samples, specifically for a type of problem, namely, the imputation of missing dynamic data in air quality monitoring stations.

This study has limitations that should be considered when interpreting the results. The analysis was conducted using data from a single station and focused on a single pollutant, which restricts the generalization of the findings to other spatial contexts and environmental variables. Furthermore, evaluation based on artificial gaps, while necessary for quantitative validation, may not fully reproduce the patterns and mechanisms associated with real missing data. As future perspectives, it is recommended to expand the study to multiple stations and pollutants, include the analysis with other exogenous variables (such as vehicle traffic flows near the monitoring station, solar radiation and rainfall index), investigate real patterns of missing data, and adopt other strategies for variable selection and hyperparameter optimization, aiming to increase the robustness, generalization, and applicability of the models in real air quality monitoring systems.

## Data Availability

The data used in this study were kindly provided by the Institute for the Environment and Water Resources (INEMA) and CETREL Environmental Protection Company Inc. and can be made available upon request.
